# Supplementary material for: Tackling immunosuppression by Neisseria gonorrhoeae to facilitate vaccine design
Source: PLoS Pathog. 2024 Nov 14;20(11):e1012688. doi: 10.1371/journal.ppat.1012688 (PMC11594432; doi:10.1371/journal.ppat.1012688)
Supplement: S1 Table — (DOCX) [file ppat.1012688.s008.docx]

**Table S1**. Primers used in this study.

| **Primer name** | **Sequence** |
| --- | --- |
| FA1090 *porB* upstream F | CGCACTGATTCAAGAACGCA |
| FA1090 *porB* upstream R | CAATCAGGGATTTTTTCATTGCTGTATTCCTTTTTTG |
| MC58 *porB* F | CAAAAAAGGAATACAGCAATGAAAAAATCCCTGATTG |
| MC58 *porB* R | ATGATGGTTGTTCAGACGGCATTTAGAATTTGTGGCGCAGACC |
| *kanR* F | GGTCTGCGCCACAAATTCTAAATGCCGTCTGAACAACCATCAT |
| *kanR* R | GCTTTTTGTTGATACCGATCTTTGCAGAAACCAATTAACCAATTCTGATTAG |
| FA1090 *porB* downstream F | CTAATCAGAATTGGTTAATTGGTTTCTGCAAAGATCGGTATCAACAAAAAGC |
| FA1090 *porB* downstream R | GGGGTAAGTTTTATCCACGACT |
| *rmpM* downstream F | CACAAACGGCATATCAAA |
| *rmpM* downstream R | ATTTAATAAGTAAGTTAAGGGATGCATAAACGGCTAGGTAATATCTTGCC |
| *rmpM* upstream F | CGTTCCGCAATATCCAAAAC |
| *rmpM* upstream R | GGTATCGGATCCAAGGGCTTTATTCCCTCATTAGATTTGTACAGCAG |
| *eryR* F | GGCAAGATATTACCTAGCCGTTTATGCATCCCTTAACTTACTTATTAAAT |
| *eryR* R | CTGCTGTACAAATCTAATGAGGGAATAAAGCCCTTGGATCCGATACC |
| *lgtE* upstream F | caacgtagaaaaatattttgcccaatc |
| *lgtE* upstream R | GCAAGACGTTTCCCGTTGAATATGGCTCATgatttatcctgttcggtttcaatagc |
| *lgtE* downstream F | TTTCATTTGATGCTCGATGAGTTTTTCTAATttgtgtaaaatataggggattaaaatcagaaatg |
| *lgtE* downstream R | tgggcatagtttttggcggtt |
