# Supplementary figures and images for: Tackling immunosuppression by Neisseria gonorrhoeae to facilitate vaccine design
Source: PLoS Pathog. 2024 Nov 14;20(11):e1012688. doi: 10.1371/journal.ppat.1012688 (PMC11594432; doi:10.1371/journal.ppat.1012688)

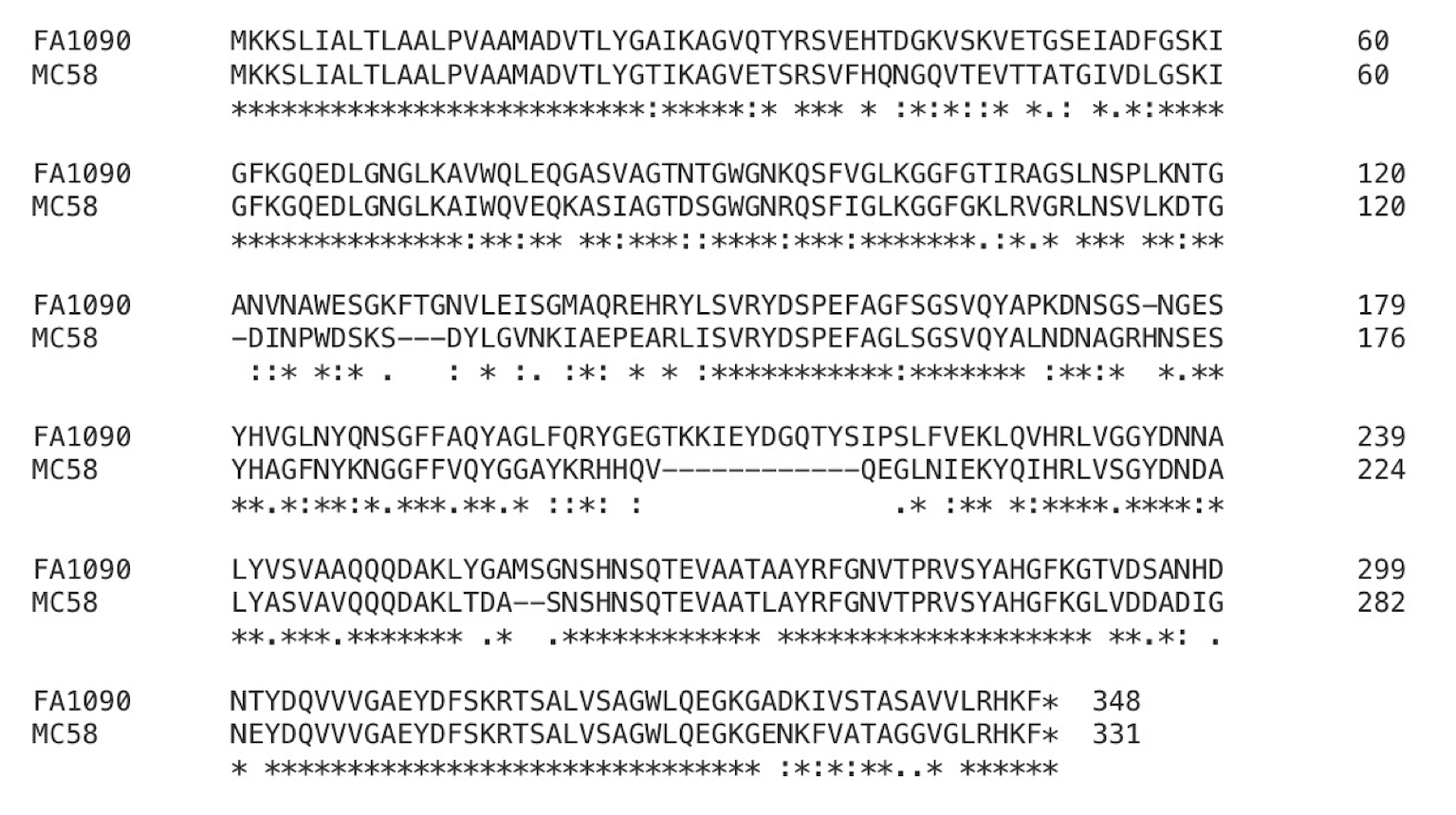

Supplement: S1 Fig — (TIFF) [file ppat.1012688.s011.tiff]

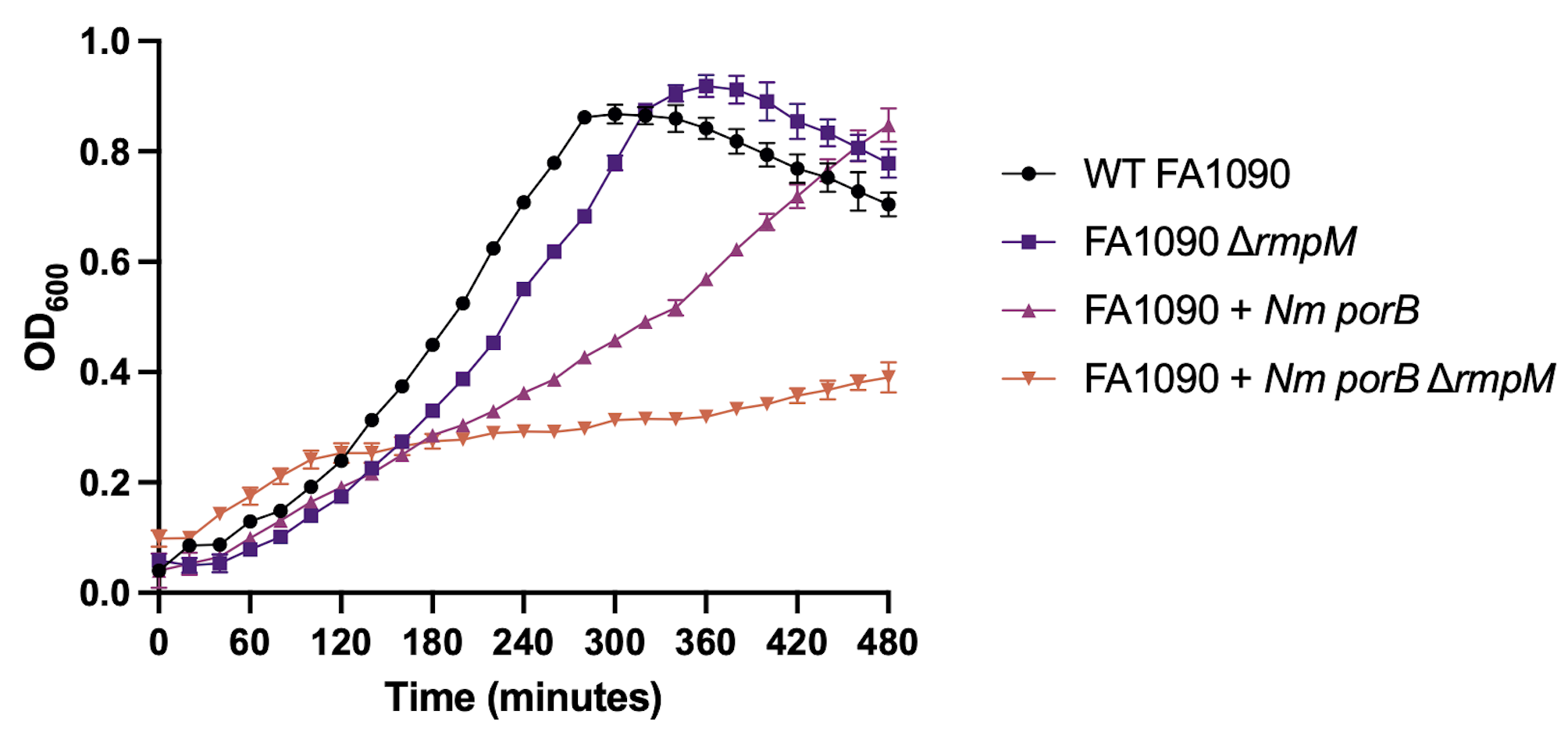

Supplement: S2 Fig — (TIFF) [file ppat.1012688.s012.tiff]

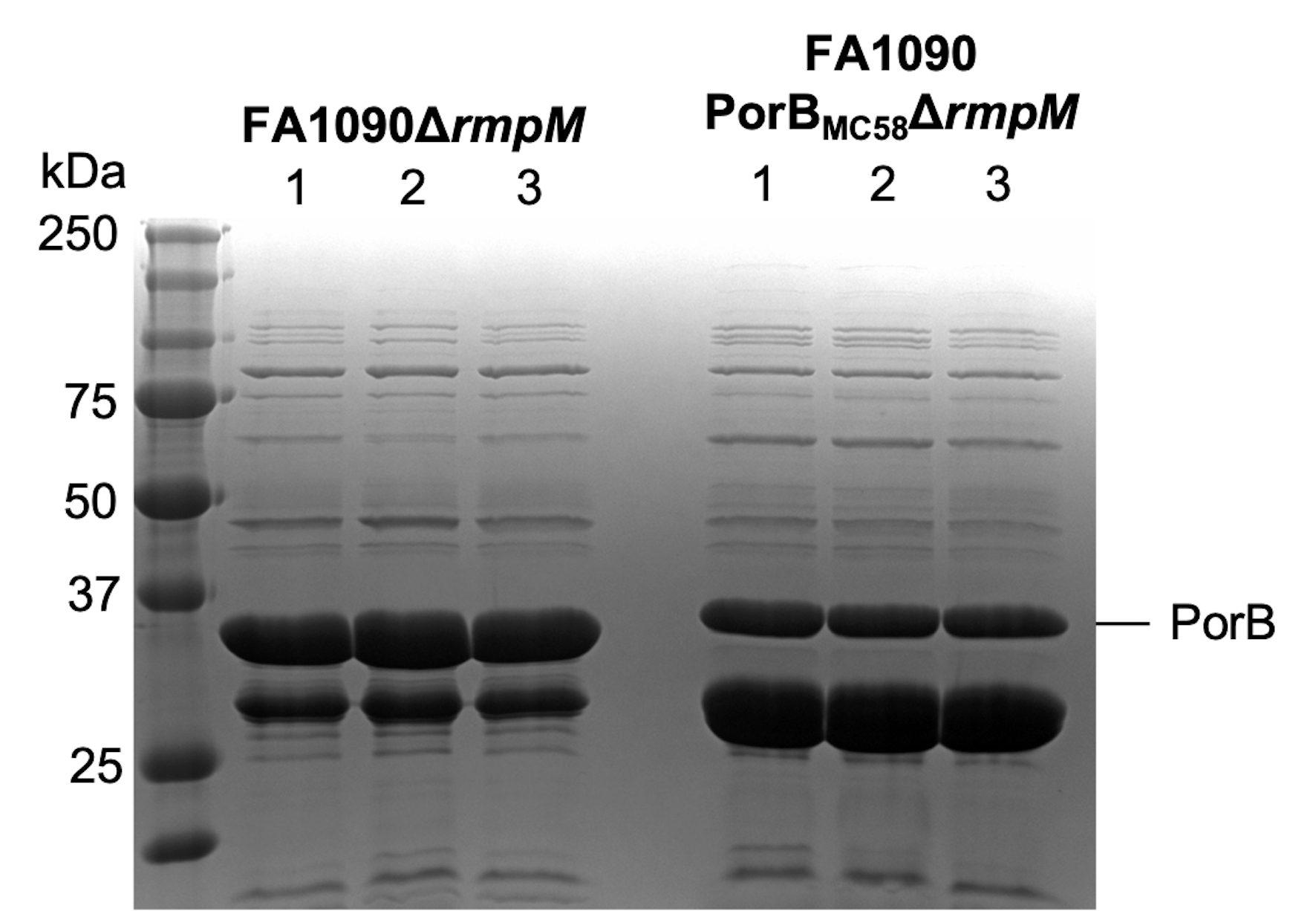

Supplement: S3 Fig — (TIFF) [file ppat.1012688.s013.tiff]

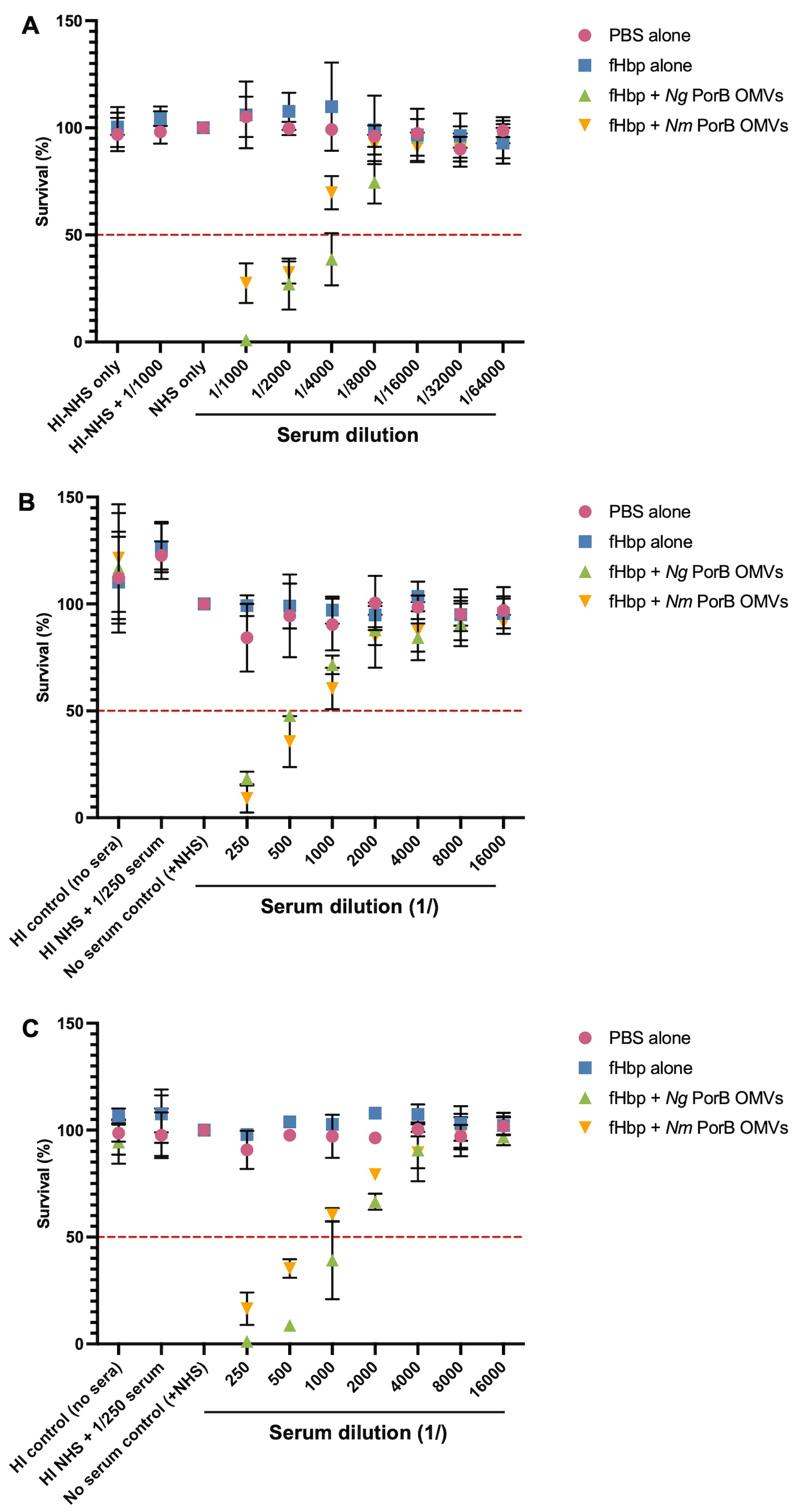

Supplement: S4 Fig — Serum bactericidal activity against WT N. gonorrhoeae FA1090 (A), G97687 (B) and 60755 (C) after immunisation with Ng- or Nm-PorB OMVs. NHS: normal human serum (IgG and IgM depleted), HI: heat inactivated. (TIFF) [file ppat.1012688.s014.tiff]

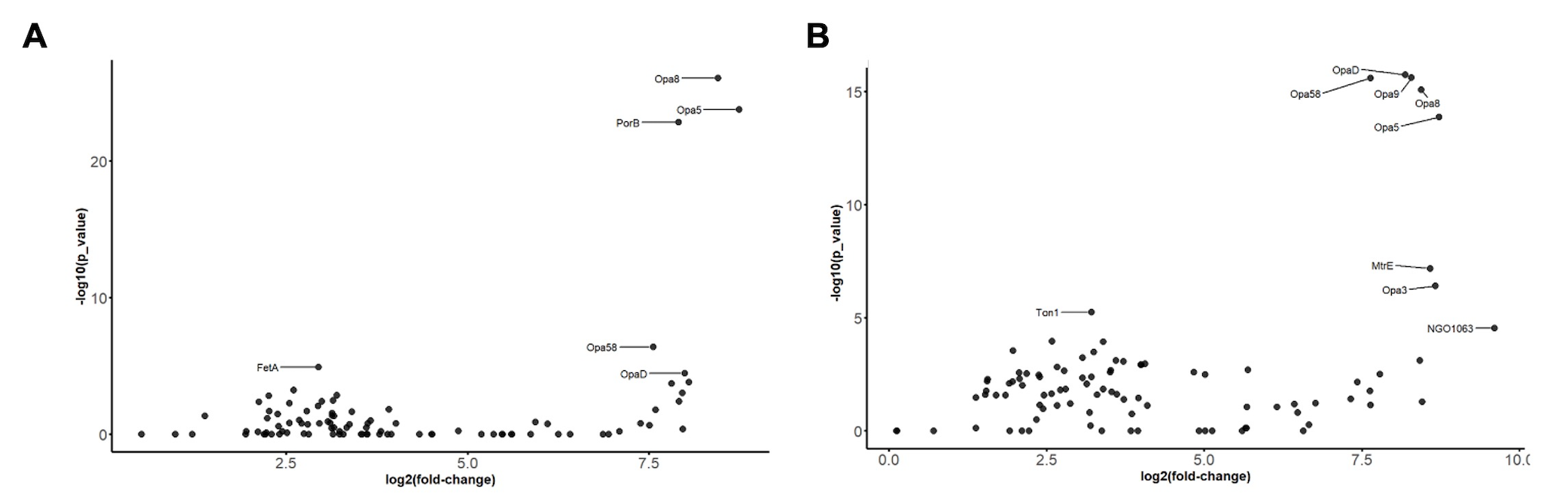

Supplement: S5 Fig — Volcano plots of total IgG responses against all antigens in the microarray A) fHbp versus Nm PorB OMVs + fHbp B) fHbp versus Ng PorB OMV + fHbp. One sided t-tests were conducted to compare IgG responses for each antigen in each paired group. The negative log10 of each p-value is plotted against log2 (binary) of the mean fold-change i.e. (mean IgG response group 2)/(mean IgG response group 1). Antigens with -log10(p-value) > 4 are labelled. (TIFF) [file ppat.1012688.s015.tiff]
