# Supplementary material for: Tackling immunosuppression by Neisseria gonorrhoeae to facilitate vaccine design
Source: PLoS Pathog. 2024 Nov 14;20(11):e1012688. doi: 10.1371/journal.ppat.1012688 (PMC11594432; doi:10.1371/journal.ppat.1012688)
Supplement: Attachment — Submitted filename: Reviewer responses_PorB swap.docx [file ppat.1012688.s016.docx]

**RESPONSES TO REVIEWERS’ COMMENTS (*in italics*)**

**Reviewer #1:**

*In this manuscript the team of authors report a novel approach to develop an outer membrane vesicle vaccine from Neisseria gonorrhoeae to prevent gonorrheal infections. Critically, they inactivated the rmpM gene, which encodes an outer membrane protein that elicits the generation of undesired and detrimental blocking antibodies; note that loss of this peptidoglycan-binding protein has been reported to increase elaboration of OMVs. Secondly, they replaced the gonococcal porB1B in strain FA1090 with the meningococcal porB gene. This is noteworthy because the gonococcal PorB1B has immunosuppressive action. The platform they describe is imaginative and worthy of further study in protection-directed experiments, which I assume is on their agenda. Although there are clear strengths to the paper I do have a few concerns, which are listed below.*

**Part II – Major Issues: Key Experiments Required for Acceptance**

**Reviewer #1:**  *1. The authors report outer membrane protein differences depending on whether the host FA1090 strain produces the Ng or Nm PorB. While I think the data are convincing it is important to note that OMVs also contain lipooligosaccharide (LOS). In this regard, the 2C7 epitope within LOS has been advanced by Rice et al. as a protective antigen. Thus, it would be good if the authors report whether this epitope is present in their OMVs and if they observed an antibody response to it.*

We thank the reviewer for the important suggestion, given that we used native OMVs that contain LOS. In additional experiments, we confirmed that both Ng and Nm PorB OMVs contain the 2C7 epitope, as expected as wild-type FA1090 contains the 2C7 epitope (Figure 4A). We also show by Western blot analysis that murine sera from Ng/Nm PorB OMV immunised mice recognise FA1090 LOS (Figure 4B). To narrow down any 2C7-specific binding was more challenging. However, we found there was a small yet reproducible reduction in LOS ELISA in the presence of the anti-2C7 monoclonal antibody (Figure 4C). Methods section: lines 819-842.

*2. The authors used SBA to ascertain whether the two OMVs preparations would generate different titers in mice. Was this done because they think it might be a correlate of protection?*

We used SBA to assess the functionality of the antibody responses we had observed (comparing Ng/Nm PorB OMVs) against wild-type intact bacteria. As we had observed differences in antibody titres against specific protein antigens, we investigated how this would translate to functionality in the form of bactericidal activity.

*A number of points need to be clarified:*

i. *what % survival do they consider as bactericidal?*
   We thank the reviewer for helping us to be clearer in our SBA data section. We have clarified that we refer to the inverse of the serum dilution that gives >50% bacterial killing as the SBA titre, and have represented the data as a table to be clearer (lines 361-386, previous figure 3 now moved to supplementary figure S4).
ii. *how many mice were used to pool immune sera?*
    We pooled sera from five mice and now describe this in the text as above, and also in the SBA Methods section (Line 878)
iii. *did they test serum from individual mice as I suspect there could be significant variation between them?*
     We only tested pooled sera
iv. *the SBA data need statistical evaluation as a two-fold difference in the titers may not be biologically important if production of bactericidal antibody is a correlate of protection;*
    We thank the reviewer for this point and agree with them. Our interpretation of the data was that the observed titres were similar between the two OMV immunised groups but we might have been not clear enough in the figure/text. We have now represented the SBA data as a table and re-written the text to present our interpretation of these data more clearly.
v. *did the pooled sera kill other Ng strains especially those producing PorB1A or antibiotic resistant strains?*
   We thank the reviewer for this interesting question. We have now also determined SBA titres on two other relevant clinical isolates; G97687 as a multi-drug resistant strain (expressing PorB.IB) from Thailand, and 60755, a PorB.IA expressing strain from Kenya (PMID: 29991383 and PMID: 29701830, respectively.). The data are reported in Table 3 (line 380), where the two OMV immunised groups showed bactericidal activity against both strains with similar SBA titres.

**Reviewer #2:**

*This study examines the possible increase in immunogenicity and potential efficacy of a gonococcal OMV vaccine that has substituted the meningococcal PorB for the gonococcal PorB. There is extensive evidence that meningococcal Porb is immuno-stimulatory via TLR2 and has been shown to act as a vaccine adjuvant. There is less evidence this property is contained within gonococcal PorB. If anything there is suggestive evidence that it could even be immuno-inhibitory They were able to show improved immunogenicity in mice with TH1 skewing and increased SBA activity.*

*Reviewer #2: Various parameters of examining the immuno-stimulatory ability of the OMV containing meningococcal PorB were examined, including IgG measurements towards a large array of gonococcal antigens, and Bactericidal activity. A significant portion of the paper was spent in explaining the construction and examination of the OMV derived from the gonococci containing NM PorB. However though immunogenicitiy was examined, by Ab levels, possible IFNg antigen specific memory recall. and SBA, other studies should be included to help truly demonstrate potential superiority to the GC OMV.*

*This would include demonstration of altered Ab binding to the native gonococcus.*

We thank the reviewer for the suggestion. We have now assessed antibody binding to whole *N. gonorrhoeae* by flow cytometry. We incubated wild-type FA1090 (the ‘parent’ OMV strain) and two clinical isolates (G97687 and 60755, see our responses to Reviewer 1) with serial dilutions of pooled OMV-immunised sera and demonstrate higher antibody deposition (indicated by fluorescence intensity) for *Nm* PorB OMV immunised mice for FA1090 and G97687; antibody binding to 60755 was similar between the two OMV-immunised groups. Figure 3G-I and text lines 318-328, and Methods lines 805-817.

*Also though there are many issues with the mouse model of infection, these studies also should be considered especially since Ann Jerse is a coauthor.*

The purpose of the present study was to determine if the immunosuppression attributed to gonococcal PorB could be mitigated. As we have promising data, we are working to finalise the vaccine platform through addition of adjuvants and/or addressing potential reactogenicity via detoxification (noted in our Discussion, lines 683-686). At this stage we consider the challenge model beyond the scope of this study.

*The splenocyte specific restimulation studies could have bene more sophisticated by purifying T cells and then use antigen presenting cells fed intact gonococci which then use these to stimulate the supposedly memory T cells induced by the vaccines. This would be instead of restimulation with the vaccine itself. This would give more definitive data and would be more relevant and related to potential gonococcal activity.*

We appreciate the comments of the reviewer. However, we have a different view of the purpose and therefore approach used in these experiments. Re-stimulation of mixed splenocytes with a vaccine antigen is commonly used to analyse cellular responses (for example, PMID: 18323851 and PMID: 10963610). This is because unfractionated splenocytes contain the necessary accessory cells including macrophages and dendritic cells that serve as antigen presenting cells that secrete secondary signals to promote T cell proliferation in the assay. Additionally, whole gonococci are known to suppress T cell proliferation by impairing antigen presentation (PMID: 22844448 and PMID: 29752412) therefore it is likely that the proposed experiment would be unsuccessful.

**Part III – Minor Issues: Editorial and Data Presentation Modifications**
Please use this section for editorial suggestions as well as relatively minor modifications of existing data that would enhance clarity.

Reviewer #1:

*The authors should indicate limitations of their work especially lack of assays that evaluated opsonophagocytic killing mediated by the pooled sera from the immunized mice.*

We have now added this to the Discussion (line 778).

Reviewer #2:

*The heat map of measurements of gonococcal IgG induced by either vaccine is hard to interpret and really doesn't display differences as compared to other possible studies like a volcano plot etc.*

As suggested by the reviewer, we have generated volcano plots for pairwise comparisons between the fHbp and Nm PorB OMV/fHbp or Ng PorB OMV/fHbp groups (Supplementary Figure S5). The plots provide information which complements the heatmaps- in the case of the fHbp versus Ng PorB OMV/fHbp comparison, PorB, Opa5 and Opa8 show the greatest stimulation in IgG and lowest p-values. For fHbp versus Nm PorB OMV/fHbp, a group of Opa proteins are identified. We also draw the attention of the reviewer to Figure 6B, which is a principal component analysis identifying the antigens that drive the separation between the two OMV immunised groups, i.e. the antigens with the largest differences in antibody responses.

*Regarding Th1 vs Th2 responses, usually a ratio of IgG1 to IgG2a is helpful in demonstration this. This is because instead of skewing, just measuring subtype levels, increases of each one may just be due to increased overall immunity instead of true skewing.*

Thank you for the comment, we have analysed IgG1/2a ratios using geometric mean titres and now present the data in Table 2 (line 315).

*The memory T cell IFNg studies would also help in this regard.*

Please see our response above.
