# Supplementary material for: Tackling immunosuppression by Neisseria gonorrhoeae to facilitate vaccine design
Source: PLoS Pathog. 2024 Nov 14;20(11):e1012688. doi: 10.1371/journal.ppat.1012688 (PMC11594432; doi:10.1371/journal.ppat.1012688)
Supplement: Attachment — Submitted filename: Jones et al cover letter.docx [file ppat.1012688.s017.docx]

Professor Christoph Tang

Professor of Cellular Pathology,

Professorial Fellow, Exeter College

tel: +44 1865 275560

email: christoph.tang@path.ox.ac.uk

17^th^ October 2024

Dear Sir/Madam,

Please find enclosed a revised version of our manuscript entitled “Tackling immunosuppression by *Neisseria gonorrhoeae* to facilitate vaccine design” to be considered for publication in *PLOS Pathogens*.

We are grateful to the reviewers for their positive comments about our manuscript and their helpful suggestions and recommendations. Reviewer 1 ‘s comments and our responses are as follows:

Reviewer #1: …. However, the new information in the text regarding the 2C7 LOS epitope is a bit confusing and does not align with the information in the legend. My reading of the section indicates that the blot probed with the 2C7 monoclonal antibody is actually shown in Figure 4A while the sera from OMV-immunized ice is shown in the two panels in 4B.

Interestingly, both of the OMV sera react with LOS from both WT and the lgtE::kan transformant (2C7-negative) suggesting that an antibody response to an antigen within the deep LOS core is possible. In this regard, I request that the authors re-evaluate their writing and make the necessary changes to improve clarity. Additionally, in future experiments it would be beneficial to use an lgtG deletion mutant to discriminate antibody responses the 2C7 epitope vs the inner region of the LOS alpha-chain given the possibility that this LOS epitope could stimulate production of bactericidal antibody.

We apologise for the incorrect labelling of Figure 4. We have rearranged Figure 4, which now reads in the order referred to in the text: 4A: Western blot of extracted LOS using OMV immunised sera, and 4B: anti-2C7 antibody, updated the legend accordingly (lines 353-359 and 915-918). As suggested, we have also now commented further on the presence of antibodies specific to a smaller LOS species (lines 334-335). We thank the reviewer for the suggestion on future experiments studying LOS responses and will consider this in future studies.

We believe the changes have improved the manuscript, and we look forward to hearing from you.

Yours
